# Ion Imbalance Is Involved in the Mechanisms of Liver Oxidative Damage in Rats Exposed to Glyphosate

**DOI:** 10.3389/fphys.2017.01083

**Published:** 2017-12-19

**Authors:** Juan Tang, Ping Hu, Yansen Li, Tin-Tin Win-Shwe, Chunmei Li

**Affiliations:** ^1^Jiangsu Province Key Laboratory of Gastrointestinal Nutrition and Animal Health, College of Animal Science and Technology, Nanjing Agricultural University, Nanjing, China; ^2^Health Effect Assessment Section Center for Health and Environmental Risk Research, National Institute for Environmental Studies, Tsukuba, Japan

**Keywords:** ion, oxidative stress, liver, glyphosate, rat

## Abstract

Glyphosate (N-phosphonomethyl-glycine, GLP) is the most popular herbicide used worldwide. This study aimed to investigate the effects of glyphosate on rats' liver function and induction of pathological changes in ion levels and oxidative stress in hepatic tissue. Sprague-Dawley rats were treated orally with 0, 5, 50, and 500 mg/kg body weight of the GLP. After 5 weeks of treatment, blood and liver samples were analyzed for biochemical and histomorphological parameters. The various mineral elements content in the organs of the rats were also measured. Significant decreases were shown in the weights of body, liver, kidney and spleen between the control and treatment groups. Changes also happened in the histomorphology of the liver and kidney tissue of GLP-treated rats. The GLP resulted in an elevated level of glutamic-oxalacetic transaminase (GOT), glutamic-pyruvic transaminase (GPT) and IL-1β in the serum. Besides, decreased total superoxide dismutase (T-SOD) activity and increased malondialdehyde (MDA) contents in the serum, liver, and kidney indicated the presence of oxidative stress. Moreover, increase of hydrogen peroxide (H_2_O_2_) level and catalase (CAT) activity in the serum and liver and decrease of glutathione (GSH) and lutathione peroxidase (GSH-Px) activity in the kidney tissue further confirmed the occurrence of oxidative stress. The results of RT-PCR showed that the mRNA expressions of *IL-1*α, *IL-1*β, *IL-6, MAPK3, NF-*κ*B, SIRT1, TNF-*α, *Keap1, GPX2*, and *Caspase-3* were significantly increased in the GLP-treated groups compared to the control group. Furthermore, *PPAR*α, *DGAT, SREBP1c*, and *SCD1* mRNA expressions were also remarkably increased in the GLP-treated groups compared to the control group. In addition, aluminum (Al), iron (Fe), copper (Cu), zinc (Zn), and magnesium (Mg) levels were showed a significant difference reduction or increase in rat liver, kidney, spleen, lung, heart, muscle, brain, and fat tissues. These results suggested that glyphosate caused obvious damage to rats' liver and caused various mineral elements content imbalances in various organs of rats. Ion imbalance could weaken antioxidant capacity and involve in the mechanism of liver oxidative damage caused by GLP.

## Introduction

Glyphosate (GLP) is a non-selective, post-emergence herbicide used for weed control in various crops, especially in rice, maize and soybean (Coutinho et al., 2005). Eighty percent of genetically modified crops were GLP-resistant plants, such as corn, soy, cotton and canola and so on (Williams et al., 2000). American farmers have widely used anti-GLP crops since 1996 (Frisvold et al., 2010). It means there will be much more glyphosate in soil and water environment. Study has reported that GLP and its metabolite such as aminomethylphosphonic acid (AMPA) and formaldehyde were found in the soil and rivers (Temple and Smith, 1992). It has been extensively demonstrated that exposure to GLP leads to oxidative stress in several tissue, including the livers and kidneys (Beuret et al., 2005; El-Shenawy, 2009; Modesto and Martinez, 2010; Larsen et al., 2012; Cattani et al., 2014).

GLP can chelate the iron (Fe) and aluminum (Al), which interferes with ion assimilation in the plant (Eker et al., 2006; Bellaloui et al., 2009). GLP also change the ion levels in fish by chelated with them (Ayoola, 2008; Samsel and Seneff, 2013). Al is widespread in soil, water, and air, and is also the most widely used metal by humans (Kumar and Gill, 2009). Al is mainly absorbed by the gastrointestinal tract and easily accumulates in liver cells and organelles (e.g., macrophages and lysosomes) (Krewski et al., 2007; Kumar and Gill, 2009). Some scholars believe that Al accumulation does not causes significant hepatotoxicity, because it can be eliminated by hepatocytes (Li et al., 2011). However, most studies reported that Al causes central nervous system toxicity, hepatotoxicity, nephrotoxicity, cardiotoxicity and osteoporosis to body tissue (Crisponi et al., 2013; Geyikoglu et al., 2013). Iron (Fe) is not only an important micronutrient, but also a redox reaction of the biocatalyst, and when the transition metal reaches the transition level, is conducive to the production of reactive oxygen species (Aust et al., 1985). Zinc (Zn) as an antioxidant, involved in cell membrane stabilization, copper/zinc superoxide dismutase (Cu/Zn SOD) structure and metallothionein induction. Zn deficiency can damage the oxidant defense system and cause oxidative damage to cells or tissue (Oteiza et al., 1999). Therefore, it is important to study whether GLP can effects the ion content in the liver and other organs of rats.

The aim of this study was designed to evaluate liver histomorphological changes, oxidant/antioxidant status, levels of inflammatory markers, lipid metabolism factors, and to investigate ion levels of Al, Fe, Cu, Zn, and Mg ion levels in GLP-exposed rats' liver tissues. The specific mechanism between liver and other organs damage and ion imbalance need to be further studied.

## Materials and methods

### Chemicals

Glyphosate, N-(phosphonomethy) glycine (GLP), was purchased from Shanghai Ryon Biological Technology Co. Ltd (Shanghai, China).

### Animals and ethic statement

Eight week-of-age male Sprague-Dawley rats weighting 180–220 g were purchased from the Nanjing Qinglongshan Experimental Animal Center (Nanjing, China). Prior to experiment, all rats were allowed to acclimate for at least 1 week. All rats were housed in separate cages under environmental conditions (23 ± 2°C, 50 ± 10% relative humidity, 12-h light: dark cycle) and had unrestricted access to food and water throughout the period of the study. Animal care and use were conducted in accordance with the National Institute of Health Guidelines for Animal Care and the Committee of Animal Research Institute, Nanjing Agricultural University, China. At the same time, the study also received ethical approval from the committee.

### Animal treatment and sample collection

Rats were randomly assigned to 4 groups (*n* = 8/group). The rats were orally administered with glyphosate (5, 50, and 500 mg/kg body weight) daily for 35 days at 9 AM. Glyphosate dose selection was according to GLP no-observed adverse effect level (NOAEL) of 1,000 mg/kg/day for developmental toxicity (Williams et al., 2000) and equivalent to 1/1,000, 1/100, and 1/10 of LD 50 in rats (Larini, 1999; Benedetti et al., 2004). GLP was orally administered at a volume of 0.5 ml/kg. Rats orally administered with distilled water were used as the control group. Twenty four hours after the last gavage, rats were weighed and decapitated. Blood samples were collected from the jugular vein and placed at 37°C for 1 h before being centrifuged (3,500 rpm, 15 min, 4°C) for biochemical assays. The liver, kidney, spleen, heart, lungs, brain, adrenal glands, muscle and fat tissue were collected, rinsed twice in phosphate-buffered saline (PBS pH 7.4), use the filter paper to dry the PBS and then accurately weigh and weighed for further examinations. One piece of liver and right kidney was used for morphometric analysis and another piece was used to prepare homogenates for analyses of tissue oxidative indexes, or frozen in liquid nitrogen for subsequent qualitative reverse transcription polymerase chain reaction (RT-PCR). The organ index is calculated as follows:

$$\begin{array}{l} \text{Organ index }\left(\text{g}/\text{gBW}\right)\\ =\text{Organ absolute weight }\left(\text{g}\right)/\text{Body weight }\left(\text{g}\right)\;\times \;100\% \end{array}$$

### Histological preparation

Samples of tissue (livers and kidneys) were obtained from the animals and fixed in 4% formaldehyde solution for 24 h then dehydrated in an ascending series of alcohol, clarified using xylene, and embedded in paraffin. Paraffin were sectioned into 5 μm slices and stained with hematoxylin-eosin (HE) for microscopic examination. The score system was used to evaluate the hepatic and renal damages (Ishak et al., 1995; Zheng et al., 2005; Klopfleisch, 2013). Briefly, the scores of liver sections graded on a 0–4 scale for lobular inflammation, focal necrosis and mononuclear cell infiltration, and kidney graded on a 0–4 scale for proximal and distal tubular necrosis, glomerular cellularity, and glomerular necrosis (where 0 represents no abnormality, and 1, 2, 3, and 4 represent mild, moderate, moderately severe, and severe abnormalities, respectively).

### Biochemical evaluation

For enzymes determination, the suspension of liver, kidney and the blood samples were centrifuged at 3,500 rpm for 15 min. The homogenate and serum were collected and used for liver function assessment including measurements of the enzymes glutamic-oxalacetic transaminase (GOT), glutamic-pyruvic transaminase (GPT), total superoxide dismutase (T-SOD), malondialdehyde (MDA), hydrogen peroxide (H_2_O_2_), catalase (CAT), glutathione (GSH), glutathione peroxidase (GSH-Px). The activities of SOD, H_2_O_2_, CAT, GSH, GSH-Px, and the content of MDA were assayed using commercial reagent kits obtained from the Institute of Biological Engineering of Nanjing Jiancheng (Nanjing, China) following the manufacturer's instructions. All operations were done at 4°C.

Analyses of the SOD activity was based on SOD-mediated inhibition of nitrite formation from hydroxyammonium in the presence of O^2−^generators (xanthine/xanthine oxidase) (Elstner and Heupel, 1976). The total SOD activity expressed as U/mg protein. MDA was evaluated by thiobarbituric acid reactive substances method (TBARS) and expressed as nmol/mg protein (Draper and Hadley, 1990). GSH-PX activity was estimated by the analysis of reduced GSH in the enzymatic reaction (Sedlak and Lindsay, 1968). GSH-PX activity was expressed as U/mg protein. CAT activity was assayed by the method developed by Aebi (Aebi, 1984), and calculated as nM H2O2 consumed/min/mg of tissue protein. Protein concentrations in the supernatant were measured according to the Coomassie Brilliant Blue method. The activity of serum GOT and GPT was assayed according to the method that usually used in clinical examination (Reitman and Frankel, 1957).

### Serum cytokine measures

Serum levels of IL-1β and IL-6 were determined using a commercially available enzyme-linked immunosorbent assay (ELISA) kit purchased from R&D Systems (Shanghai, China). The results were expressed as pg/mL.

### Quantitative RT-PCR (qRT-PCR) analysis

Total RNA was extracted from the tissue using the reagent box of Total RNA Kit (Invitrogen, Carlsbad, CA, US), according to the manufacturer's instructions. The concentration of RNA was measured by using a spectrophotometer and the purity was ascertained by the A 260/A 280 ratio with a Nanodrop® 8000. Total RNA from each sample was reverse transcribed to cDNA with an Omniscript® Reverse Transcription kit (Takara) with Oligo-dT primers (Takara) according to the manufacturer's instructions and used for RT-PCR. The target fragments were quantified by real-time PCR using a QuantiTectTMSYBR Green® PCR Kit (Roche) with 100 ng of the cDNA template. Each sample was tested in duplicate. The gene expression data were normalized to β-actin expression. The primers used correspond to the rat sequences shown in Table 1; primer design was done using Amplify software (TaKaRa, Nanjing, China). For each real-time PCR assay, the threshold cycle Ct was determined for each reaction. Ct values for each gene of interest were normalized to the housekeeping gene (β-action); PCR amplification efficiencies were taken into account by amplifying various amounts of target cDNA for each reaction. The fold differences in mRNA expression of samples were relative to the internal control sample, which was included in all runs.

**Table 1:** Primers used for quantitative real-time PCR.

**Gene symbol**	**Accession No**.	**Primer sequence (5′to 3′)**	**Product size (bp)**	**40 PCR cycles**
IL-1α	NM_017019.1	F: GAGTCGGCAAAGAAATCAAGA	112	95°C for 15 s 60°C for 30 s 72°C for 30 s
		R: TTCAGAGACAGATGGTCAATGG		
IL-1β	NM_031512.2	F: GCCAACAAGTGGTATTCTCCA	120	95°C for 15 s 60°C for 30 s 72°C for 30 s
		R: TGCCGTCTTTCATCACACAG		
IL-6	NM_012589.2	F: AGTTGCCTTCTTGGGACTGA	102	95°C for 15 s 60°C for 30 s 72°C for 30 s
		R: ACTGGTCTGTTGTGGGTGGT		
MAPK3	NM_017347.2	F: CTACACGCAGCTGCAGTACATC	153	95°C for 15 s 60°C for 30 s 72°C for 30 s
		R: GTGCGCTGACAGTAGGTTTGA		
NF-kB	NM_001276711.1	F: CGACGTATTGCTGTGCCTTC	198	95°C for 15 s 60°C for 30 s 72°C for 30 s
		R: TTGAGATCTGCCCAGGTGGTA		
SIRT1	NC_005119.4	F: GAAACCCTCAATTTCTGTTCTGCT	226	95°C for 15 s 60°C for 30 s 72°C for 30 s
		R: AATGCGATGCTGACTTCCTTCT		
TNF-α	NM_012675.3	F: TTCCGTCCCTCTCATACACTG	149	95°C for 15 s 60°C for 30 s 72°C for 30 s
		R: AGACACCGCCTGGAGTTCT		
Keap1	NM_057152.2	F: CATCGGCATCGCCAACTTC	278	95°C for 15 s 60°C for 30 s 72°C for 30 s
		R: GCTGGCAGTGTGACAGGTTGA		
GPx2	NM_183403.2	F: CCGTGCTGATTGAGAATGTG	113	95°C for 15 s 60°C for 30 s 72°C for 30 s
		R: AGGGAAGCCGAGAACCACTA		
Caspase-3	NM_012922	F: AAGCCGAAACTCTTCATC	349	95°C for 15 s 60°C for 30 s 72°C for 30 s
		R: TGAGCATTGACACAATACAC		
PPARα	NM_001145367.1	F: CTCGTGCAGGTCATCAAGAA	158	95°C for 15 s 60°C for 30 s 72°C for 30 s
		R: CAGCCCTCTTCATCTCCAAG		
DGAT	NM_053437.1	F: TCTTCCTACCGGGATGTCAATC	204	95°C for 15 s 60°C for 30 s 72°C for 30 s
		R: TCCCTGCAGACACAGCTTG		
SREBP1c	NM_001271207.1	F: GCCATGGATTGCACATTG	187	95°C for 15 s 60°C for 30 s 72°C for 30 s
		R: TGTGTCTCCTGTCTCACCCC		
SCD1	NM_009127.4	F: CCTTAACCCTGAGATCCCGTAGA	237	95°C for 15 s 60°C for 30 s 72°C for 30 s
		R: AGCCCATAAAAGATTTCTGCAAA		
FAS	NM_139194.2	F: GGACATGGTCACAGACGATGAC	279	95°C for 15 s 60°C for 30 s 72°C for 30 s
		R: GGAGGCGTCGAACTTGGA		
β-actin	NM_031144.3	F:AGCCATGTACGTAGCCATCC	227	95°C for 15 s 60°C for 30 s 72°C for 30 s
		R:CTCTCAGCTGTGGTGGTGAA		

### Ion concentration

The concentrations of Al, Fe, Cu, Zn, and Mg in the liver, kidney, spleen, lung, heart, muscle, brain, and fat tissue were determined by inductively coupled plasma optical emission spectrometry (Optima 2100 DV; Perkin Elmer, Waltham, MA) using nitric acid–perchloric acid–based wet digestion. Approximately 200 μl or 0.5 g of each sample was digested with nitric acid (75%) and perchloric acid (25%) in a microwave digester (MDS- 81D; CEM Corp., Matthews, NC). We have used the same part of organ from the control and treated animals and accurately weighed.

### Statistical analysis

The data were expressed as mean ± standard error of the mean (SEM) and were analyzed by one-way analysis of variance (ANOVA), followed by Dunnett's multiple comparison tests, which was performed with GraphPad Prismsoftware (GraphPad Software, San Diego, CA, USA). Differences were considered to be statistically significant when the *p* level was less than 0.05.

## Results

### Body and organ weights

After administration of GLP, there was a significant distinction in rat body weight between the control group and the 500 mg/kg GLP group (*p* < 0.05, Table 2). The body weight gain decreased significantly in 50 mg/kg and 500 mg/kg GLP treatment groups compared with the control group (*p* < 0.05). Significant difference was also observed in the average-day-gain and average daily feed intake in GLP treatment groups compared with the control group (*p* < 0.05). Both of the absolute organ weight or the relative organ weight for liver, spleen and kidney showed a significant decrease in the 500 mg/kg GLP group (*p* < 0.05, Table 2), which suggested that GLP manifest toxicity principally toward growth and development at the studied dosages.

**Table 2:** Body weights and organ weights of rats treated with Glyphosate for 5 weeks.

	**Control**	**GLP (mg/kg body weight)**		
	**0**	**5**	**50**	**500**
Number of animals	8	8	8	8
Initial body weight (g)	298.60 ± 5.17	323.30 ± 4.94	313.40 ± 7.12	311.40 ± 8.87
Body weight (g)	388.60 ± 7.08	404.00 ± 5.71	369.30 ± 12.57	351.80 ± 7.74^*^
Weight gain percentage (%)	30.40 ± 3.18	23.42 ± 1.06	17.38 ± 2.49^**^	17.29 ± 5.41^*^
Average daily gain (g)	2.57 ± 0.25	2.09 ± 0.10	1.72 ± 0.20^*^	1.49 ± 0.17^*^
Average daily feed intake (g)	3.16 ± 0.05	3.27 ± 0.05	2.86 ± 0.05^**^	2.98 ± 0.08
Liver (g)	12.94 ± 0.45	12.98 ± 0.36	11.83 ± 0.74	10.66 ± 0.44^*^
Relative liver (%)	3.51 ± 0.07	3.30 ± 0.10	3.20 ± 0.21	2.95 ± 0.09^*^
Spleen (g)	0.77 ± 0.04	0.71 ± 0.03	0.76 ± 0.06	0.59 ± 0.04^*^
Relative Spleen (%)	0.21 ± 0.01	0.18 ± 0.01	0.20 ± 0.01	0.16 ± 0.01^*^
Kidney (g)	1.19 ± 0.04	1.27 ± 0.03	1.18 ± 0.07	1.00 ± 0.02^*^
Relative Kidney (%)	0.33 ± 0.01	0.32 ± 0.01	0.31 ± 0.01	0.29 ± 0.01^*^
Heart (g)	1.29 ± 0.05	1.40 ± 0.08	1.22 ± 0.12	1.16 ± 0.07
Relative Heart (%)	0.34 ± 0.01	0.36 ± 0.02	0.34 ± 0.04	0.33 ± 0.02
Lung (g)	2.33 ± 0.08	2.60 ± 0.12	2.40 ± 0.17	2.30 ± 0.10
Relative Lung (%)	0.61 ± 0.02	0.64 ± 0.03	0.69 ± 0.06	0.66 ± 0.03
Adrenal (g)	0.033 ± 0.002	0.034 ± 0.003	0.036 ± 0.003	0.038 ± 0.004
Relative Adrenal (%)	0.009 ± 0.001	0.009 ± 0.001	0.011 ± 0.001	0.011 ± 0.001

The values shown are the mean ± SEM of 8 animals per group. Compared to control;

* *p < 0.05*,

** *p < 0.01*.

### Histopathologic evaluation

**Figure 1:**
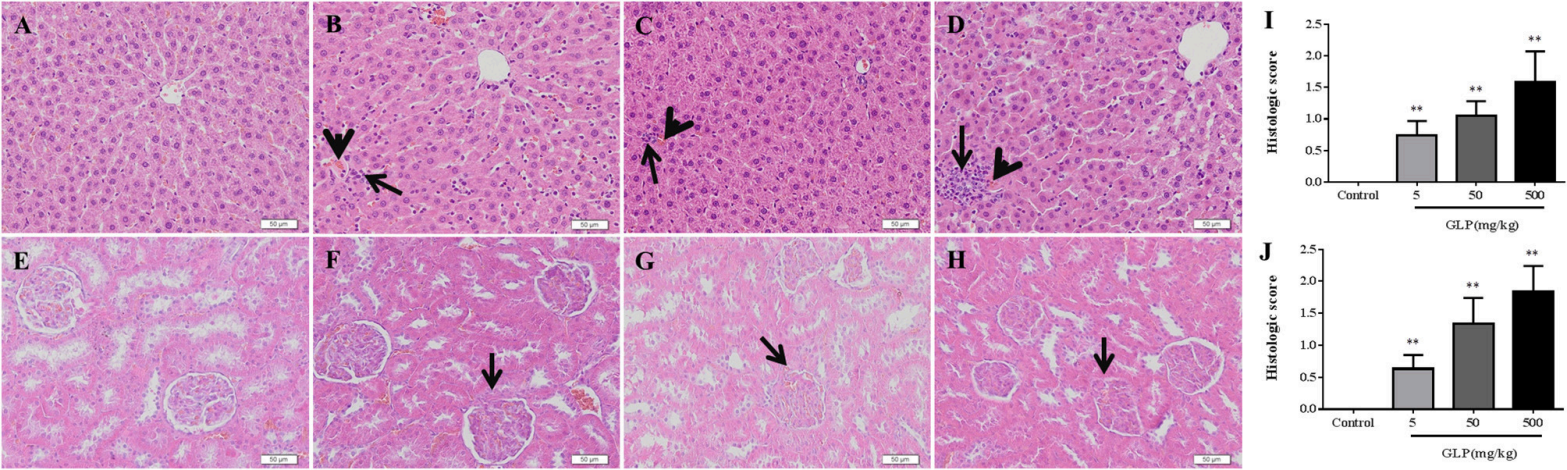
Histopathological changes in the livers and kidneys of male rats following oral GLP administration through Hematoxylin and eosin staining. 200 × magnification. **(A)** Normal liver section. Hepatic lobules consisting of a central vein surrounded by radiating hepatocytes which were separated; **(B–D)** GLP (5, 50, and 500 mg/kg/day) treated presenting periportal expansion, structural disorder, monocyte infiltration (arrows), and congestion (arrowheads). **(E)** Normal kidney section. No signs of kidney damage were observed in the kidney of controls; **(F–H)** GLP (5, 50, and 500 mg/kg/day) treated presenting proximal and distal tubular necrosis and glomerular toxicity (arrows). **(I,J)** The hepatic and renal damages histologic score evaluating. Data shown are mean ± SEM of six liver sections in each group. Compared to control; ^**^p < 0.01.

The liver and kidney histopathological changes were showed in Figure 1. The control rats showed hepatic lobules consisting of a central vein surrounded by radiating hepatocytes which were separated and did not exhibit any damage in the tissue (Figure 1A). By contrast, the liver sections of GLP-treated rats showed apoptosis of some hepatocyte, focal necrosis and mononuclear cell infiltration in liver tissue. Compared with the control group, after 5 mg/kg of GLP exposure, the rats showed mild periportal expansion and apoptosis of some hepatocyte (Figure 1B). In comparison, the livers of rats in the 50 mg/kg and 500 mg/kg GLP-treated groups demonstrated greater levels of structural disorder, apoptosis of some hepatocyte and monocyte infiltration (Figures 1C,D).

The HE staining of renal tissue in control rats demonstrated overall integrity of glomerulus surrounded by Bowman capsule and convoluted tubules (Figure 1E). In comparison with control kidney, GLP administration induced markable histological changes, including proximal and distal tubular necrosis and glomerular toxicity (Figures 1F–H). And the histologic score of hepatic and renal damages was significantly increased in the both GLP-treated groups compared with the control group (*p* < 0.01) (Figures 1I,J).

### Assessment of liver function

**Figure 2:**
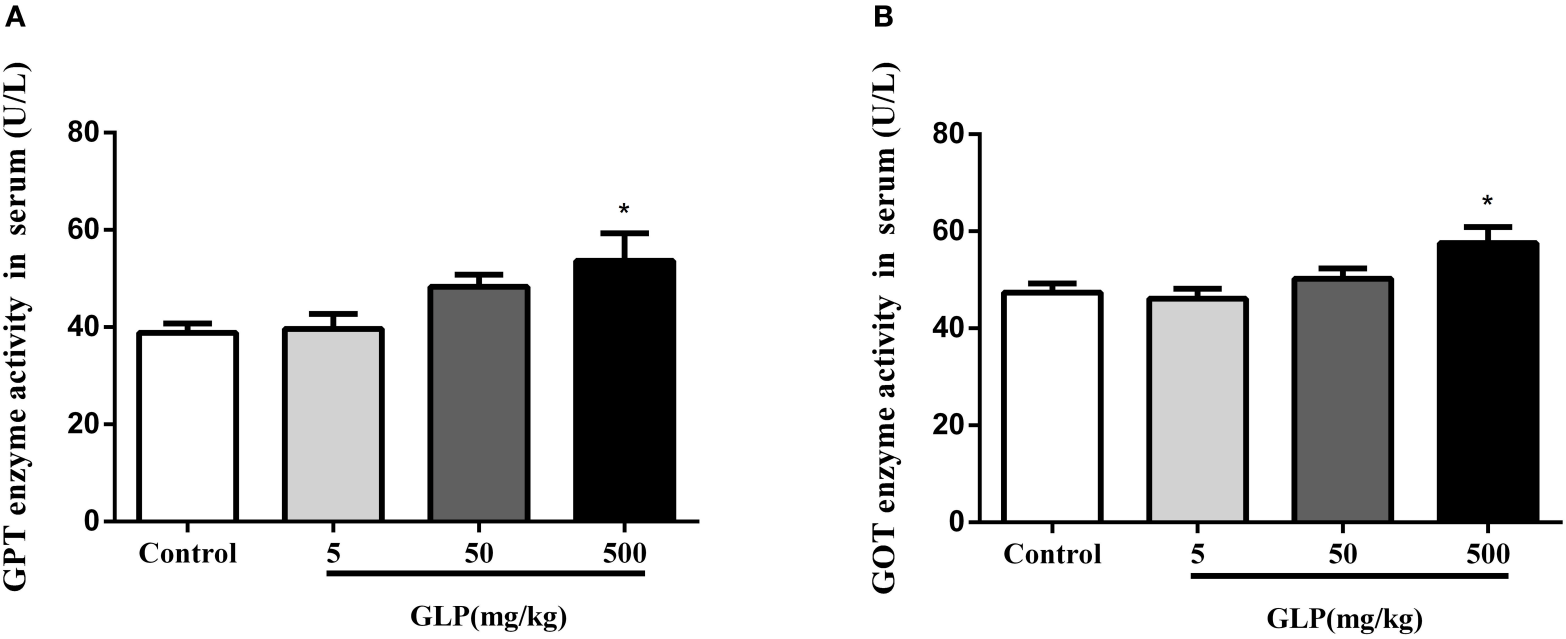
Effect of GLP treatment on GPT **(A)** and GOT **(B)** enzyme activities in the serum. Data shown are mean ± SEM of eight animals in each group. Compared to control; ^*^p < 0.05.

To confirm the damage of GLP to liver, the serum GOT and GPT levels, the main enzymes of liver function, were determined. The results showed that the levels of the GOT and GPT were increased in GLP-treated groups compared with the control rats. Furthermore, there was a significant increase in GOT and GPT levels with 500 mg/kg of glyphosate compared with the control group (*p* < 0.05) as shown in Figures 2A,B. These results showed that glyphosate can affect hepatic metabolism, causing oxidative damage to the hepatic tissue.

### Assessment of enzyme levels in the serum to test oxidative stress

To determine whether the GLP could induce the oxidative stress *in vivo*, we first examined the SOD, CAT, GSH, and GSH-PX activities as well as the level of MDA in the serum. The results showed that SOD activity significantly decreased in the 500 mg/kg GLP-treated group compared to the control (*p* < 0.05). The MDA content showed significant increase in the 50 mg/kg GLP-treated group compared with the control (*p* < 0.05), and significantly increased CAT activity than the control in the 500 mg/kg GLP-treated group compared with the control (*p* < 0.05) (Table 3).

**Table 3:** Effects of GLP on antioxidant enzyme activities and lipid peroxidation levels in serum, liver, and kidney of rats.

	**Control**	**GLP (mg/kg body weight)**		
	**0**	**5**	**50**	**500**
**SERUM**				
SOD (U/mL)	13.29 ± 0.16	13.01 ± 0.58	12.89 ± 0.46	11.30 ± 0.28^*^
MDA (nmol/mL)	20.72 ± 2.16	22.06 ± 2.03	32.38 ± 2.00^**^	23.23 ± 1.99
H_2_O_2_ (nmol/mL)	113.50 ± 10.05	113.80 ± 8.87	117.50 ± 6.63	134.30 ± 7.45
CAT (U/mL)	28.33 ± 1.61	28.66 ± 2.22	30.51 ± 1.52	36.56 ± 1.60^*^
GSH (mg/L)	536.30 ± 22.76	431.90 ± 46.45	446.20 ± 52.51	423.40 ± 47.15
GSH-PX (U/L)	378.20 ± 37.47	400.80 ± 28.74	429.00 ± 37.64	453.00 ± 13.76
**LIVER**				
SOD (U/mgprot)	49.77 ± 2.06	50.30 ± 2.32	47.08 ± 1.49	41.53 ± 1.19^*^
MDA (nmol/mgprot)	1.93 ± 0.06	1.89 ± 0.08	1.86 ± 0.08	2.08 ± 0.07
H_2_O_2_ (nmol/mgprot)	5.10 ± 0.26	5.42 ± 0.27	5.90 ± 0.23	6.27 ± 0.14^*^
CAT (U/mgprot)	12.04 ± 0.68	13.01 ± 1.61	14.50 ± 1.22	14.48 ± 1.20
GSH (mg/gprot)	316.40 ± 27.98	342.20 ± 26.16	272.90 ± 25.01	357.80 ± 33.52
GSH-PX (U/mgprot)	64.09 ± 5.76	63.90 ± 7.62	56.43 ± 7.82	46.74 ± 4.39
**KIDNEY**				
SOD (U/mgprot)	97.67 ± 4.51	87.62 ± 6.51	75.10 ± 4.17^*^	73.06 ± 3.31^*^
MDA (nmol/mgprot)	1.87 ± 0.20	3.91 ± 0.25^**^	3.015 ± 0.49	3.026 ± 0.40
H_2_O_2_ (nmol/mgprot)	5.28 ± 0.34	6.03 ± 0.63	6.68 ± 0.46	5.58 ± 0.37
CAT (U/mgprot)	764.20 ± 38.35	873.70 ± 66.49	865.90 ± 83.28	819.60 ± 54.81
GSH (mg/gprot)	7.70 ± 1.85	4.31 ± 0.45	2.76 ± 1.06^*^	4.64 ± 0.75
GSH-PX (U/mgprot)	480.00 ± 19.96	421.80 ± 38.47	342.20 ± 40.60^*^	297.00 ± 36.08^**^

The values shown are the mean ± SEM of 8 animals per group. Compared to control;

* *p < 0.05*,

** *p < 0.01*.

### Assessment of enzyme levels in the liver and kidney to test oxidative stress

Liver and kidney are two major organs that suffer from the oxidative stress, since GLP metabolism mainly occurs in the liver and the metabolites discharge in the kidney. After GLP exposure, SOD activity in the 500 mg/kg GLP-treated group showed significant decrease in the liver compared with the control (*p* < 0.05). However, the level of H_2_O_2_ in the 500 mg/kg GLP-treated group significantly increased compared with the control group (*p* < 0.05) (Table 3).

Next, the activity of antioxidant enzymes in the kidney was examined. As shown in Table 3, the MDA content in the 5 mg/kg GLP-treated group showed significant increase compared with the control group (*p* < 0.01). The SOD and GSH-PX activities were significantly decreased in the 500 mg/kg GLP-treated groups compared with the control group (*p* < 0.05). And the GSH activity also showed significant decrease in the 50 mg/kg GLP-treated groups compared with the control group (*p* < 0.05). However, there was no difference for the H_2_O_2_ and CAT activities between the control and treatment groups (Table 3).

### Serum IL-1β and IL-6 levels

**Figure 3:**
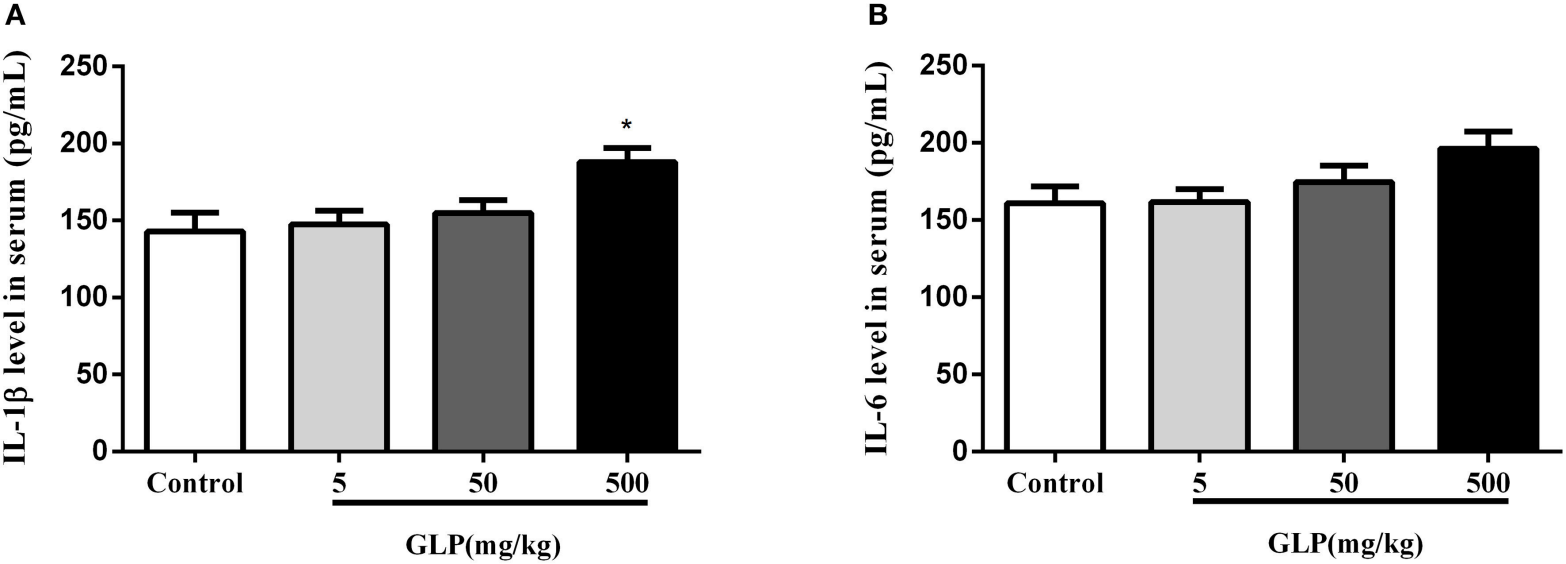
The level of IL-1β **(A)** and IL-6 **(B)** in the serum was assayed by ELISA. Data shown are mean ± SEM of eight animals in each group. Compared to control; ^*^p < 0.05.

The concentrations of inflammatory mediators IL-1β and IL-6 in serum were determined as shown in the Figure 3. The level of IL-1β has a significant increase in the 500 mg/kg GLP-treated group compared with the control rats (*p* < 0.05) (Figures 3A,B).

### Expression of mRNA levels for inflammation related genes in the liver

**Figure 4:**
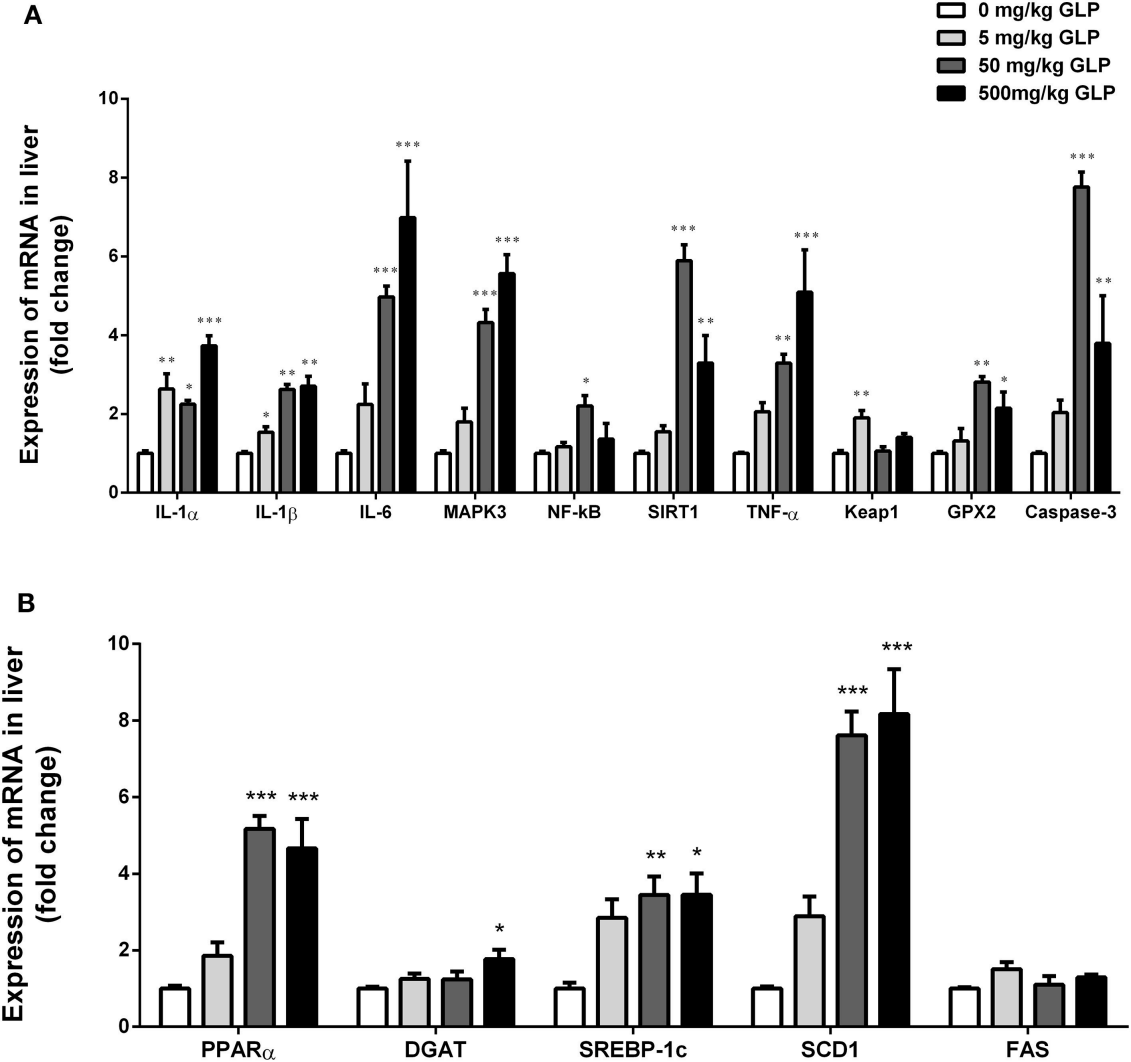
Real-time RT-PCR analyses of IL-1α, IL-1β, IL-6, MAPK3, NF-kB, SIRT1, TNF-α, Keap1, GPX2, and Caspase-3 mRNA of liver **(A)**. Real-time RT-PCR analyses of PPARα, DGAT, SREBP1c, SCD1, and FAS mRNA of liver **(B)**. Data shown are mean ± SEM of eight animals in each group. Compared to control; ^*^p < 0.05, ^**^p < 0.01 and ^***^p < 0.001.

We investigated the effects of GLP involved in the inflammatory response in the liver tissue (Figure 4A). Hepatic *IL-1*α *and IL-1*β mRNA expression were significantly increased after GLP exposure compared with the control group (*p* < 0.05); *IL-6, MAPK3, SIRT1, TNF-*α, *GPX2*, and *Caspase-3* mRNA expression were significantly increased in the 50 mg/kg and 500 mg/kg GLP-treated group compared with the control group (*p* < 0.05); *NF-*κ*B* mRNA expression showed a significant increase in the 50 mg/kg GLP-treated group compared with the control group (*p* < 0.05); at the same time, we also observed a significant increase in *Keap1* mRNA expression in 5 mg/kg GLP-treated group compared with the control group (*p* < 0.05).

### Expression of mRNA levels for lipid metabolism related genes in the liver

Compared with the control group, *PPAR*α, *SREBP1c*, and *SCD1* mRNA expression were significantly increased in the 50 mg/kg and 500 mg/kg GLP treatment rats (*p* < 0.05); *DGAT* mRNA expression was significantly increased in the 500 mg/kg GLP-treated group compared with the control group (*p* < 0.05) (Figure 4B).

### Concentrations of ions in liver, kidney, spleen, heart, lung, brain, muscle, and fat

Concentrations of Al, Fe, Cu, Zn, and Mg in the liver, kidney, spleen, lung, heart, muscle, brain and fat were presented in Tables 4, 5.

**Table 4:** The concentrations of Al, Fe, Cu, Zn, and Mg in the liver, kidney, spleen, and heart of rats.

	**Control**	**GLP (mg/kg body weight)**		
	**0**	**5**	**50**	**500**
**LIVER**				
Al (mg/kg)	1.75 ± 0.13	2.22 ± 0.14	2.88 ± 0.33^*^	3.14 ± 0.45^*^
Fe (mg/kg)	189.00 ± 12.56	244.0 ± 13.60^*^	218.10 ± 10.44	233.70 ± 13.64
Cu (mg/kg)	8.47 ± 0.13	8.14 ± 0.32	8.06 ± 0.15	8.64 ± 0.30
Zn (mg/kg)	63.45 ± 0.86	63.12 ± 3.46	72.24 ± 1.70^*^	73.46 ± 2.24^*^
Mg (mg/kg)	614.30 ± 12.87	579.60 ± 23.19	592.10 ± 11.77	608.90 ± 18.79
**KIDNEY**				
Al (mg/kg)	2.20 ± 0.37	2.52 ± 0.15	2.62 ± 0.33	2.53 ± 0.62
Fe (mg/kg)	150.80 ± 5.64	163.10 ± 7.22	216.70 ± 25.09^*^	151.00 ± 16.76
Cu (mg/kg)	11.15 ± 0.73	11.49 ± 0.34	11.45 ± 0.43	11.09 ± 0.52
Zn (mg/kg)	59.15 ± 3.53	58.93 ± 2.70	62.55 ± 1.61	56.06 ± 2.18
Mg (mg/kg)	593.90 ± 24.84	605.30 ± 27.81	669.20 ± 20.71	582.40 ± 15.86
**SPLEEN**				
Al (mg/kg)	10.27 ± 0.66	10.05 ± 1.09	9.37 ± 0.83	10.31 ± 0.68
Fe (mg/kg)	1162.00 ± 208.00	1704.00 ± 230.30	2049.00 ± 188.10^*^	1466.00 ± 98.26
Cu (mg/kg)	7.45 ± 0.35	7.15 ± 0.50	7.76 ± 0.35	7.16 ± 0.15
Zn (mg/kg)	117.10 ± 2.53	120.30 ± 11.39	111.60 ± 7.23	112.50 ± 4.94
Mg (mg/kg)	1185.00 ± 34.60	1182.00 ± 26.26	1240.00 ± 45.99	1159.00 ± 29.09
**HEART**				
Al (mg/kg)	7.07 ± 0.59	7.97 ± 0.37	7.43 ± 0.43	7.30 ± 1.04
Fe (mg/kg)	321.30 ± 11.96	367.70 ± 37.90	401.20 ± 27.08	313.20 ± 25.61
Cu (mg/kg)	20.58 ± 0.79	18.74 ± 0.27	18.63 ± 0.88	18.44 ± 0.47
Zn (mg/kg)	88.82 ± 5.52	79.85 ± 2.45	82.80 ± 5.42	78.39 ± 10.11
Mg (mg/kg)	1131.00 ± 24.37	1059.00 ± 40.66	1039.00 ± 56.45	987.80 ± 33.74

The values shown are the mean ± SEM of 8 animals per group. Compared to control;

* *p < 0.05*.

**Table 5:** The concentrations of Al, Fe, Cu, Zn, and Mg in the lung, brain, muscle and fat of rats.

	**Control**	**GLP (mg/kg body weight)**		
	**0**	**5**	**50**	**500**
**LUNG**				
Al (mg/kg)	26.55 ± 1.14	21.93 ± 1.05^*^	20.43 ± 0.71^**^	23.27 ± 1.52
Fe (mg/kg)	362.30 ± 31.16	446.00 ± 32.30	421.40 ± 35.80	647.30 ± 74.77^**^
Cu (mg/kg)	10.31 ± 0.27	10.13 ± 0.46	8.92 ± 0.40	9.94 ± 0.83
Zn (mg/kg)	211.00 ± 14.07	236.50 ± 14.07	187.10 ± 6.99	244.90 ± 20.62
Mg (mg/kg)	1133.00 ± 17.40	1114.00 ± 18.28	1045.00 ± 21.90	1016.00 ± 63.47
**BRAIN**				
Al (mg/kg)	4.74 ± 1.16	5.08 ± 0.73	5.38 ± 0.55	4.17 ± 0.24
Fe (mg/kg)	40.79 ± 0.59	50.21 ± 6.04	47.38 ± 2.20	53.01 ± 1.49
Cu (mg/kg)	4.08 ± 0.04	4.50 ± 0.06	5.35 ± 0.59	5.70 ± 0.24^*^
Zn (mg/kg)	35.59 ± 1.36	37.82 ± 1.08	51.28 ± 8.54	39.62 ± 1.71
Mg (mg/kg)	357.60 ± 8.83	357.90 ± 12.81	415.40 ± 3.05^**^	416.50 ± 6.58^**^
**MUSCLE**				
Al (mg/kg)	7.52 ± 0.55	7.54 ± 0.44	6.56 ± 0.54	5.48 ± 0.20^*^
Fe (mg/kg)	52.76 ± 2.45	57.89 ± 6.47	54.62 ± 4.48	72.62 ± 22.37
Cu (mg/kg)	3.68 ± 0.27	3.47 ± 0.24	3.51 ± 0.35	3.46 ± 0.15
Zn (mg/kg)	53.05 ± 4.89	53.28 ± 2.37	55.79 ± 3.83	58.50 ± 6.08
Mg (mg/kg)	1313.00 ± 14.13	1197.00 ± 26.59	1262.00 ± 33.24	1223.00 ± 55.23
**FAT**				
Al (mg/kg)	3.22 ± 0.29	2.37 ± 0.57	4.09 ± 0.40	3.23 ± 0.15
Fe (mg/kg)	11.16 ± 1.33	10.92 ± 4.01	16.44 ± 2.33	14.41 ± 1.82
Cu (mg/kg)	0.29 ± 0.05	0.35 ± 0.10	0.53 ± 0.03^*^	0.61 ± 0.04^**^
Zn (mg/kg)	5.65 ± 0.81	5.35 ± 0.53	6.36 ± 0.70	6.27 ± 1.02
Mg (mg/kg)	25.49 ± 1.25	25.99 ± 2.60	33.41 ± 1.96	33.57 ± 4.12

The values shown are the mean ± SEM of 8 animals per group. Compared to control;

* *p < 0.05*,

** *p < 0.01*.

In liver, compared with the control group, Al and Zn concentrations were significantly increased in 50 mg/kg and 500 mg/kg GLP treatment group (*p* < 0.05); Fe concentration was significantly increased in 5 mg/kg GLP treatment group (*p* < 0.05) and Mn concentration was significantly increased in 500 mg/kg GLP treatment group (*p* < 0.05); Mo concentration was significantly decreased in 5 mg/kg and 50 mg/kg GLP treatment group (*p* < 0.05) (Table 4).

In kidney, concentrations of Fe level was significantly increased in 50 mg/kg GLP treatment group compared with the control group (*p* < 0.05) (Table 4).

In spleen, Fe content showed significant increase in 50 mg/kg GLP treatment group compared with the control group (*p* < 0.05) (Table 4).

In lung, Al concentration was significantly decreased in 5 mg/kg and 50 mg/kg GLP treatment groups compared with the control group (*p* < 0.05); Fe concentration was significantly increased in 500 mg/kg GLP treatment group compared with the control group (*p* < 0.05) (Table 5).

In brain, Cu content was significantly increased in 500 mg/kg GLP treatment group compared with the control group (*p* < 0.05); Mg concentration significantly increased in 50 mg/kg and 500 mg/kg GLP treatment group compared with the control group (*p* < 0.05) (Table 5).

In muscle, Al concentration was significantly decreased in 500 mg/kg GLP treatment group (*p* < 0.05) (Table 5).

In fat tissue, concentrations of Cu was significantly increased in 50 mg/kg and 500 mg/kg GLP treatment groups compared with the control group (*p* < 0.05) (Table 5).

## Discussion

The present study demonstrated that GLP had an adverse effect on the histomorphology, inflammation, oxidative stress, lipid metabolism and ion concentration in adult male rats, and then discussed the relationship between them. This is the first report about the effects of GLP exposure on Al, Fe, Cu, Zn, and Mg content in main tissues of rats. Also we firstly revealed the connection between dysregulation of ion content and liver injury in rats exposed to GLP.

The results of our study showed that exposure to GLP for 35 days led to a significant reduction in body weight, body weight gain, average daily gain, and liver, spleen and kidney coefficient. These results suggested that treated with GLP in male rats for 35 days could affect the growth performance of rats. In addition, our results also showed that exposure to GLP for 35 days caused significant hyperemia, cellular degeneration and necrosis accompanied inflammatory cell infiltration, renal tubular damage and glomerular filtration impairment in rats' hepatic and kidney cells, accompanied by significant increases in GPT and GOT levels. Transaminases are important enzymes and critical enzymes in the biological processes. GPT and GOT levels increased in serum can be a sign of liver damage and disruption of normal liver function (El-Demerdash et al., 2001; Celik and Suzek, 2008). Results showed that GLP caused damage in liver morphology and function.

Oxidative stress refers to the oxidation and anti-oxidation imbalance *in vivo* (Hou et al., 2013). Some studies reported that GLP is an organophosphate herbicide and can induce to oxidative stress and/or an impairment of the antioxidant defensive mechanisms (Larsen et al., 2012). Animals possess an antioxidant defense mechanism composed of enzymes including T-SOD and GPx, as well as non-enzymatic antioxidants including non-protein thiols, especially GSH. When the defenses of the organism are insufficient for neutralizing the ROS, oxidative damage can occur, and one of the most serious types of which is membrane lipid peroxidation (Ahmad et al., 2004). Liver is the major detoxification organ exposed to food or drinks contaminants (Gasnier et al., 2009). GLP-based herbicide has been demonstrated to damage carp or rat hepatocytes at low levels (Szarek et al., 2000; Malatesta et al., 2008).

MDA, the stable metabolite of lipid peroxidation (LPO) products, is a biomarker of LPO (Sun et al., 2001), and is presented as the total level of LPO products (Drewa et al., 2002). MDA can be produced by ozone, which reacts rapidly with cellular structures and generates hydrogen peroxide (Ajamieh et al., 2004). Hepatic SOD activity also can suggest the extent of liver damage (Li et al., 2013). CAT catalyzing the breakdown of H_2_O_2_ into O_2_ and H_2_O and catalyzing the oxidation of electron donors (Hou et al., 2013). In addition, GSH provide the major defense against oxidative stress induced cellular damage (Beuret et al., 2005; Ozden and Alpertunga, 2010). In the present study, our results showed that SOD activity significantly decreased in the serum, liver and kidney of the GLP-treated rats compared with the control group. MDA content showed significant increase in the serum and kidney of the GLP-treated rats. At the same time, CAT activity was also significantly increased in the serum of the GLP-treated rats compared with the control group. In addition, H_2_O_2_ increased in the liver tissue, suggesting *t* that rats were under the oxidant stress. Taken together, the data demonstrated that GLP could result in liver and kidney damage, the decreased SOD activity in the serum and tissue, and the increased MDA level in the serum, indicative of oxidative stress. On the other side, we have also tested the inflammatory Cytokines level in serum, our results showed that the level of IL-1β has a significant increase in the 500 mg/kg GLP-treated group compared with the control rats. Thus, we investigate whether the oxidative stress state of organism has a certain relationship with the inflammation related genes.

Inflammation, manifested as macrophage infiltration of adipose tissue, endoplasmic reticulum stress and oxidative stress (Trayhurn and Wood, 2004). In a few cases, steatosis causes apoptosis, necrosis, generation of oxidative stress and inflammation (Marchesini et al., 2008). Animal models of nonalcoholic fatty liver disease have also suggested a possible role of free fatty acids, not triglycerides, in the hepatocytes as factors promoting hepatocellular injury (Yamaguchi et al., 2007). GLP induced inflammation, which was found to be associated with induction of IL-33, which is known to induce TNF-α, IFN-γ, and IL-13 upon antigen challenge followed by activation and recruitment of inflammatory cells in the airways (Kumar et al., 2014). In this study, the mRNA expression of *IL-1*α, *IL-1*β, *IL-6, MAPK3, NF-*κ*B, SIRT1, TNF-*α, *Keap1, GPX2* and *Caspase-3* were all increased in GLP treatment group compared with the liver tissue of control rats. Meanwhile, *PPAR*α, *SREBP1c, DGAT*, and *SCD1* mRNA expressions were significantly increased in GLP treatment rats. It showed that GLP induced liver toxicity is mediated by inflammation, oxidative stress and lipid related pathways. In addition, in the present study, we only focus on changes in inflammatory markers and lipid metabolite levels in the liver, possible changes in kidneys will continue to be verified in future experiments.

Additionally, previous studies also indicated that GLP is bound to the soil constituent Fe, Al amorphous hydroxides and ferric oxides (Piccolo et al., 1994; Day et al., 1997). GLP negatively impact human health, and interference with cytochrome P450 (CYP) enzymes, which play many important roles in the body, meanwhile, GLP chelation of minerals, such as iron and cobalt (Samsel and Seneff, 2013). Al accumulation resulted in obvious damage to hepatic cells, including liver central venous hyperemia, lipid accumulation, and lymphocyte infiltration (Bogdanović et al., 2008; Türkez et al., 2010). Fe is an essential nutritional mineral for all life forms, both of Fe deficiency and excess in Fe also leads to oxidative DNA damage (Ames, 2001). Becaria reported that Al augmented oxidative stress injuries induced by Fe (Becaria et al., 2002). Zn has a relationship with many enzymes in the body (Powell, 2000; Ozturk et al., 2003; Ozdemir and Inanc, 2005). One study has shown that Zn deficiency increases lipid peroxidation in various rat tissues (Ozdemir and Inanc, 2005). Mg plays a pivotal role as an enzyme cofactor in biosynthesis of proteins and mineral administration. It is indispensable to osteogenesis and mineralization of bones (Rahnama and Marciniak, 2002). Subacute Mg deficiency can cause lymphopoietic neoplasms in young rats (Ilicin, 1971). Mg, Zn, and Cu are the cofactors of SOD. Fe and Cu overload could cause oxidative stress damage to rats' kidney and liver (Ozcelik et al., 2003; Bishu and Agarwal, 2006). This study results showed that the concentration of Al, Fe and Zn were significantly increased in GLP-treated rats' liver. Concentrations of Fe were also increased in the kidney, spleen, and lung tissue in GLP-treated rats. Al concentration was decreased in the muscle tissue of GLP-treated rats. In brain and fat tissue, Cu and Mg concentration were increased in GLP-treated rats. However, there showed no dose-dependent effect of GLP was found. Combined, these results suggested that GLP induced the ion-imbalance of Al, Fe, Mg, Cu, and Zn, which will make damage to hepatic cells and liver dysfunction, and the role of ion-imbalance in renal and other organs will continue to be verified in future experiments.

In summary, current study demonstrated that GLP causes obvious damage to rat liver, kidney and caused ion-imbalance in main tissue of rats, and the ion-imbalance is no dose-dependent effect of GLP was found. It may be due to the too large dose range we used in the study of the GLP. Ion imbalance-related oxidative stress may be involved in the mechanism of chronic liver injury caused by GLP. Simultaneously, GLP-induced ion imbalance and oxidative stress may also affect kidney damage. Therefore, the role of ion-imbalance in renal and other organs and its mechanism must be further confirmed by systematic experiments in the future.

## Author contributions

JT, PH, YL, T-TW-S, and CL: Performed experiments and interpreted data; CL: Designed the study and provided funding; JT: Wrote the manuscript. All authors read and approved the final version of the manuscript.

### Conflict of interest statement

The authors declare that the research was conducted in the absence of any commercial or financial relationships that could be construed as a potential conflict of interest.
